# Thyroid Stimulating Hormone, T3 and T4 Population-based Reference Range and Children Prevalence of Thyroid Dysfunction: First Report from South of Iran

**DOI:** 10.30699/IJP.2022.541736.2812

**Published:** 2022-09-13

**Authors:** Mansoureh Shokripour, Mohammad Hadi Imanieh, Saghar Garayemi, Navid Omidifar, Babak Shirazi Yeganeh, Fadhil Althabhawee

**Affiliations:** 1 Department of Pathology, School of Medicine Shiraz University of Medical Sciences, Shiraz, Iran; 2 Gastroenterohepatology Research Center, Namazi Hospital, School of Medicine, Shiraz University of Medical Sciences, Shiraz, Iran; 3 Clinical Education Research Center, School of Medicine, Shiraz University of Medical Sciences, Shiraz, Iran

**Keywords:** Iran, Range, Reference, Reference range, TSH

## Abstract

**Background & Objective:**

The TSH reference range's validity affects the thyroid dysfunction diagnosis. The primary objective of this study is to determine the reference range, which is established according to age and region.

**Methods:**

The data were collected retrospectively from people over the age of one who visited Motahari Clinic for routine health checkups between August 2017 and October 2019. TSH, T4, T3, personal drug usage, and thyroid history were collected. After excluding subjects with thyroid diseases and outliers, 1392 participants were analyzed. Hormone intervals of men and women ≥1 year old have been determined using the non-parametric method.

**Results:**

The non-disease subjects' TSH, T3, and T4 reference ranges were 0.64 to 5.94 lU/mL, 0.91 to 2.47 ng/dL, and 5.53 to 12.48 g/dL, respectively. According to this range, total thyroid dysfunction prevalence in our study in children was 8.94%. There was no significant difference between TSH, T4 level, and gender in the non-disease population (*P*=0.46 and 0.13, respectively), but there was a statistical difference between sex and T3 (*P* =0.03). Our study also illustrates that for subjects under 18 years old and older, the hormones (TSH, T3, T4) concentration are statistically different (*P*≤0.001).

**Conclusion:**

We found a statistically difference between hormone values younger and older than age 18 (*P*=≤0.01); therefore, it is not appropriate to use the same reference range for children younger than age 18 and adults. There was male predominance in the population aged1-18 years old.

## Introduction

The thyroid gland is responsible for adjusting metabolic rate of the body (1). Two essential hormones, thyroxine and triiodothyronine, frequently called T4 and T3, are excreted by the thyroid gland. The development of the fetus and fetal central nervous system depends on thyroid hormones (2, 3). Another hormone called thyroid-stimulating hormone (TSH) adjusts the amount of hormone production by the thyroid (4). The functional thyroid condition is assessed by the TSH hormone made by the pituitary gland, which is the most delicate marker for this purpose. TSH aids in identifying subjects who suffer from thyroid malfunction. Subclinical hypo- and hyperthyroidism are defined as increased and decreased TSH, respectively, with normal thyroid gland hormones (5). The increased awareness of thyroid ailments and health checkups is causing increased incidences of subclinical hypo- and hyperthyroidism (6). 

A reference range helps physicians make a medical or therapeutic administration decision or other physical assessment based on a person's observed laboratory test outcome (usually a patient) (7). Analysis of laboratory records is a relational decision-making procedure; as a result, we need reference values for all tests in the clinical laboratory to improve this decision-making process. Providing consistent reference ranges is important for clinical laboratories and diagnostic test companies. The most frequently used reference values (also known as "normal values" or "expected values") have historically been inadequately defined and not determined by a consistent procedure (8). It is apparent that it is key to establish reference ranges via a process that considers the various impacts on the measured laboratory test outcomes (8). Forming a reference range of TSH is critical in the clinical setting to find subclinical thyroid functional disorders (9, 10).

Although the prevalence of hypothyroidism in Iran is unknown (11), studies show that congenital hypothyroidism is a common health problem in Iran (12, 13). A thyroid function test composed of TSH, T4, T3, free T4 (FT4), and free T3 (FT3) is used to detect clinical ailments of the thyroid gland. Such tests should have normal ranges, which helps distinguish an unhealthy patient from a healthy one. So, it is crucial to make this suitable information accessible to physicians (14).

Studies have revealed that TSH's median and upper levels decline with increasing age (15, 16). Using the same reference range of TSH for all ages has increased the over-diagnosis of hypo- or hyperthyroidism, suggesting that levothyroxine treatment in the older population is redundant.

Different factors such as lifestyle, race, age/developmental stage, gender, diet, and other environmental elements affect reference ranges; it is evident that for every population in different sections, reference ranges should be established (17). Many factors, such as diurnal variations and even seasonal changes, may affect the results of laboratory tests (18). National Academy of Clinical Biochemistry suggested that the serum TSH normal range should be carefully created for each laboratory from selected normal subjects without signs of thyroid disease (19).

In our study, we suggest reference ranges according to age and gender for T3, T4, and TSH and the prevalence of thyroid defectives in subjects aged 1- 18 years old in the Motahari Clinic in Shiraz, a referral center in the south of Iran. In addition, we assessed the relationship between TSH, T3, T4, and gender and the differences in hormone values according to age groups in this population.

## Material and Methods

**Subjects' Selection**

The data were gathered retrospectively from those over age one who came in for a routine health checkup at Motahari Clinic from August 2017 to October 2019 in a cross-sectional study of the population. The subjects underage one were eliminated due to the slight number of cases in this age range. The history of the research subjects was completed via a telephone questionnaire, which was administered by an experienced person. Before the interview, they were informed that their sample information was used for research, and the nature of the research was explained to them. The interview was done if they agreed. 

**Laboratory Methods**

As the routine protocol of the laboratory, nearly 10 mL of blood have been sampled for the examination of serum TSH, T3, and T4 in each patient. The blood samples were taken in a sitting position and the morning when the patient has been fasting for at least 2 hours. The samples were conveyed to the testing center, and then the serum was separated within 30 minutes. Samples were analyzed by radioimmunoassay (RIA) within 12 hours. 

Hormone volumes were measured using a kit, with reference ranges of 0.3-6.1 lU/mL,0.5-2.5 ng/dL and 4.5-13.5 µg/dL for TSH, T3, and T4, respectively.

**Non-Disease Populations and Total Population Definitions**

Subjects with no family history and no previous history of thyroid dysfunction, and no drug history that could impact thyroid behavior, were considered non-disease population. The total population was demarcated as all the subjects in the telephone interview. It is worth mentioning that the women who were pregnant were excluded from the study.

**Prevalence of Thyroid Dysfunctions for Children aged 1-18**

We reported the prevalence of subclinical and overt hypo- and hyperthyroidism in different age groups of 1-18 years old according to the current definition. We gathered the results of 1708 subjects in the mentioned age range and calculated prevalence for different age and sex categories according to hormone reference ranges obtained from our study based on the non-disease population.

**Statistical Analysis**

Statistical assessment was accomplished by SPSS version 25 (SPSS Inc., Chicago, Ill., USA). TSH, T3, and T4 reference ranges were compared in seven age groups within two gender categories. The histogram, Q-Q plot, and Kolmogorov-Smirnov (K-S) Test of data distribution were used for the graphical and numerical determination, respectively. The data distribution does not follow a Gaussian distribution (*P*≤0.001). As a result, we use the non-parametric method (Kruskal-Wallis test) to evaluate the relations between hormone values and demographic variables (sex, age). So, the 2.5th and 97.5th percentiles were reported to establish the reference range for our studied hormones. According to IFCC and CLSI, for such sort of data, we can use n=(100/p)-1 to find the minimum individuals required per partition (8). In our study, the number of subjects in each age range is more than or equal to 39 (For the age range where the number of data was <40, we report the maximum of the data as the 97.5th percentile). 

A P-value less than 0.05 was considered significant. The median (Inter Quartile Range [IQR]) was calculated as descriptive statistics.

## Results

Patients' Baseline Characteristics

**Fig. 1:**
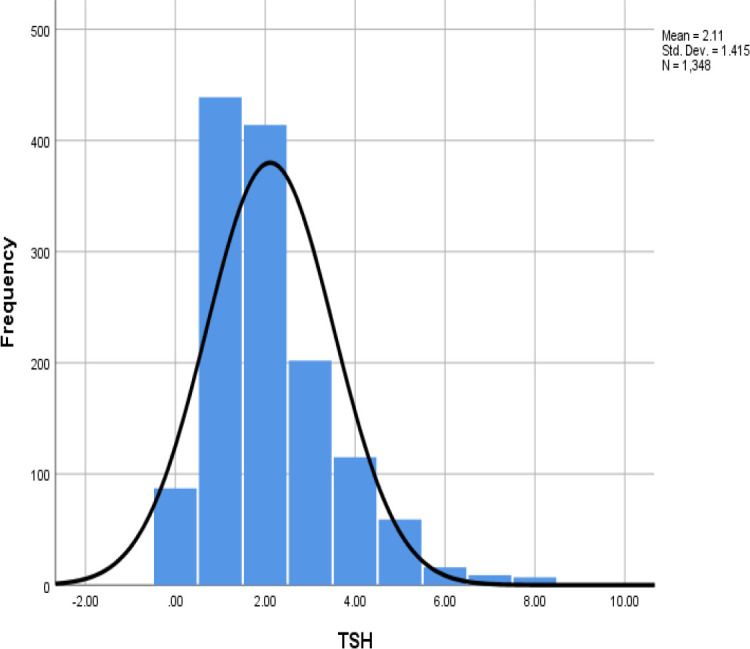
Frequency of the TSH values

The Thyroid function test was adapted for the whole 1392 subjects in our study. Among them, 548 were males (39.36%), 844 were females (60.63%), and the median age was 20. Table 1 presents the distribution of each age group. Skewness was present in the overall distribution of TSH (Figure 1), with skewness and kurtosis of 1.65 and 5.26, respectively. 

TSH, T3, and T4 Concentrations

The median serum TSH level was 2.33 µlU/mL. The 2.5th and 97.5th percentiles of TSH levels were 0.63 and 6.29 µlU/mL, respectively (Table 2). The median TSH amount in the non-disease subjects was 2.32 µlU/mL (2.38 µlU/mL for males and 2.29 µlU/mL for females). The 2.5th percentile of TSH levels was 0.64 µlU/mL (men subjects 0.6 µlU/mL, women subject 0.73 µlU/mL) and the 97.5th percentile of TSH was 5.94 µlU/mL (men 5.90 µlU/mL women 6.01 µlU/mL). Age-related reference ranges for TSH are shown in Table 3.

The median T4 level was 8.57 µg/dL. The 2.5th and 97.5th percentiles of T4 were 5.59 and 12.5 µg/dL, respectively (Table 2). In the non-disease subjects, the median of T4 was 8.55 µg/dL (men and women 8.63 and 8.45 µg/dL, respectively). We found that the 2.5th percentile of T4 as the lower limit of reference range was 5.53 µg/dL (men 5.27 µg/dL, women 5.6 µg/dL) and the 97.5th percentile of T4 as the upper limit of reference range was 12.48 µg/dL (men 12.57 µg/dL women 12.41 µg/dL). Age-related reference ranges for T4 are shown in Table 4.

We also evaluated the median of T3 for our non-disease subjects. It was 1.5 ng/dL (1.55 for men and 1.47 ng/dL for women). The 2.5th percentile of T3 was 0.91 ng/dL (men 0.92 ng/dL, women 0.88 ng/dL). The 97.5th percentile of T3 was 2.47 ng/dL (men 2.45 ng/dL women 2.41 ng/dL). Age-related reference ranges for T3 are shown in Table 5.

**Prevalence of Hypothyroidism for the Age Range 1-18**

Prevalence of overt and subclinical hypothyroidism was 0.17% and 5.91%, respectively (Table 6). The prevalence of subclinical hypothyroidism was 7.06% in males and 4.97% in females. The prevalence of overt hypothyroidism in men and women was 0.26% and 0.1%, respectively.

Association of Hormone Concentration and Baseline Characteristics

Dividing our sample into two sub-samples, under 18 years old and above, illustrates that hormone (TSH, T3, T4) concentration is statistically different in these two age categories (*P*≤0.001). No statistical difference was found between TSH, T4 level, and sex in the non-disease population (*P*=0.46 and 0.13, respectively). Still, there was a statistical difference between sex and T3 (*P*=0.03, men have a greater value of T3). A statistical test also determined that for subjects aged 1-18 distribution of hormones concentration was the same between males and females and we derived the same result for subjects older than 18 (*P*>0.05). Women and men under 18 do not have a significant difference in TSH, T3, and T4 concentration (*P*=0.70, 0.56, 0.96, respectively). We derived the same result for males and females older than 18 (*P*=0.74, 0.58, 0.56, respectively). Significant statistical associations with their P-values for other age subgroups are shown in Table 7.

**Table 1:** Baseline characteristic of the subjects who underwent thyroid function tests

Total population									
						
	**TSH**			**T3**			**T4**		
Age, yr	Total	Men	Women	Total	Men	Women	Total	Men	Women
1-3	145(10.4)	81(14.8)	64(7.6)	142(10.3)	80 (14.7)	62(7.5)	141(10.4)	79 ( 14.6)	62(7.6)
4-6	176 (12.6)	91(16.6)	85(10.1)	176(12.8)	91(16.7)	85)10.2)	173 (12.7)	91 (16.9)	82(10)
7-10	191(13.7)	92(16.8)	99(11.7)	186(13.5)	89 (16.4)	97(11.7)	185 (13.6)	89 (16.5)	96(11.7)
11-18	83 (6)	41(7.5)	42(5)	83(6)	41 (7.5)	42(5.1)	82 (6)	40(7.4)	42(5.1)
19-29	555(39.9)	146(26.6)	409(48.5)	547(39.8)	146(26.8)	401(48.2)	541(39.8)	145(26.9)	396(48.3)
30-50	140(10.1)	45(8.2)	95(11.3)	140(10.2)	45(8.3)	95(11.42)	138 (10.1)	45(8.3)	93(11.4)
>50	102 (7.3)	52(9.5)	50(5.9)	102(7.4)	52(9.6)	50(6)	100(7.4)	51(9.5)	49(9)
aNon-disease subjects									
						
	**TSH**			**T3**			**T4**		
Age, yr	Total	Men	Women	Total	Men	Women	Total	Men	Women
1-3	135(10)	75(5.6)	60(4.5)	132(9.9)	74(14)	58(7.2)	132(10)	74(14.1)	58(7.3)
4-6	167 (12.4)	87(6.5)		80(5.9)	167(12.5)	87 (16.5)	80(9.9)	164(12.4)	87(16.6)
7-10	171(12.7)	86 (6.4)	85(6.3)	167(12.5)	86(16.3)	83(10.31)	166(12.6)	84(16)	82(10.3)
11-18	79 (5.9)	40(3)	39(2.9)	79(5.9)	40(7.6)	39(4.9)	78(5.9)	39(7.4)	39(49)
19-29	554 (41.1)	146(10.8)	408(30.3)	546(41)	146 (27.7)	400(49.7)	541(41)	145(27.6)	396(49.9)
30-50	140(10.4)	45(3.3)	95(7)	140(10.5)	45(8.5)	95(11.8)	138(10.5)	45(8.6)	93(11.7)
>50	102(7.6)	52(3.9)	50 (3.7)	102(7.7)	52(9.9)	50 (6.2)	100(7.6)	51(9.7)	49(6.1)

a Subjects with no family history and no previous history of thyroid dysfunction and no drug history that could impact thyroid behavior were considered as non-disease subjects.

**Table 2:** Serum TSH, T3 and T4 hormone concentration in all and the non-disease subjects

Total population	Non-disease subjects					
TSH, µlU/mL	Total (n=1392)	Men (n=548)	Women (n=842)	Total (N=1348)	Men (N=531)	Women (N=817)
2.5^th ^percentile	0.63	0.6	0.71	0.64	0.6	0.73
50^th^ percentile	2.33	2.39	2.29	2.32	2.38	2.28
97.5^th^ percentile	6.29	7.10	6.10	5.94	5.91	6.01
T3,ng/dL	n=1376	n =544	n =832	N=1333	(N=528)	(N=805)
2.5^th ^percentile	0.91	0.92	0.88	0.91	0.92	0.88
50^th^ percentile	1.50	1.55	1.47	1.5	1.55	1.47
97.5^th^ percentile	2.47	2.48	2.47	2.47	2.49	2.41
T4,µg/dL	N=1360	N=820	N=540	N=1319	(N=525)	(N=794)
2.5^th ^percentile	5.59	5.31	5.63	5.53	5.27	5.6
50^th^ percentile	8.57	8.63	8.51	8.55	8.63	8.455
97.5^th^ percentile	12.50	12.59	12.46	12.48	12.57	12.41

**Table 3:** Thyroid-stimulating hormone concentration by age and gender (µIU/mL)

Total subjects											Non-disease subjects								
Total					Men			Women			Total			Men			women		
Percentile					Percentile			percentile											
Group	Age,yr	2.5	50	97.5	2.5	50	97.5	2.5	50	97.5	2.5	50	97.5	2.5	50	97.5	2.5	50	97.5
1	1-3yr	0.79	2.34	7.06	0.49	2.37	9.39	0.78	2.33	5.83	0.75	2.33	5.36	0.457	2.33	5.18	0.77	2.33	5.88
2	4-6yr	0.83	2.52	7.56	0.65	2.6	7.77	0.88	2.46	6.99	0.86	2.48	7.61	0.63	2.59	7.79	1.13	2.44	7.05
3	7-10yr	0.54	2.74	8.16	0.43	2.89	8.25	0.64	2.48	8.42	0.56	2.65	6.04	0.40	2.89	6.71	0.93	2.43	6
4	11-18yr	0.42	2.48	6.26	0.45	2.2	5.86	0.04	2.48	8.75	0.47	2.48	6.29	0.45	2.12	5.89	0.47	2.4	8.96
5	19-29yr	0.75	2.27	5.54	0.75	246	5.62	0.74	2.23	5.86	0.76	2.28	5.54	0.75	2.46	5.62	0.76	2.23	5.51
6	30-50yr	0.39	2.15	6.67	0.29	1.91	6.02	0.27	2.24	5.56	0.39	2.15	6.67	0.24	2.03	5.56	0.27	2.24	7.31
7	>50	0.49	1.93	5.81	0.67	1.94	5.49	0.29	1.91	6.02	0.49	1.93	5.81	0.67	1.94	5.49	0.29	1.91	6.02

**Table 4:** T4 hormone concentration by age and gender (µg/dL)

Total population											Non-disease subjects								
Total					Men			Women			Total			Men			women		
Percentile					Percentile			percentile			percentile			percentile			percentile		
Group	Age, yr	2.5	50	97.5	2.5	50	97.5	2.5	50	97.5	2.5	50	97.5	2.5	50	97.5	2.5	50	97.5
1	1-3yr	3.05	8.89	12.65	1.84	8.77	13.4	4.72	9	12.28	2.90	8.91	12.58	1.79	8.74	12.8	4.37	9.17	12.33
2	4-6yr	5.28	9.47	12.83	3.78	9.3	12.52	6.75	9.58	13.45	5.08	9.51	12.89	3.68	9.3	12.54	6.66	9.86	13.60
3	7-10yr	6.57	8.87	13.23	6.41	8.88	13.58	6.31	8.86	13.37	6.38	8.74	13.54	6.36	8.98	13.67	6.06	8.71	13.57
4	11-18yr	5.27	8.35	11.90	4.96	8.07	11.18	5.50	8.61	16.91	5.25	8.21	11.70	4.96	8.10	11.19	5.41	8.33	11.92
5	19-29yr	5.56	8.42	12.40	5.18	8.65	12.69	5.6	8.33	12.39	5.56	8.42	12.40	5.1	8.65	12.69	5.6	8.33	12.39
6	30-50yr	3.24	8.09	11.48	1.84	8.33	12.01	2.88	7.99	11.86	3.24	8.09	11.84	1.84	8.33	12.01	2.88	7.99	11.86
7	>50	5.83	7.96	11.85	5.57	7.82	11.96	5.74	8.23	11.81	5.83	7.96	11.85	5.57	7.82	11.96	5.74	8.23	11.81

**Table 5:** T3 hormone concentration by age and gender (ng/dL)

Total population											Non-disease subjects								
Total					Men			Women			Total			Men			Women		
Percentile					Percentile			Percentile			Percentile			Percentile			Percentile		
Group	Age,yr	2.5	50	97.5	2.5	50	97.5	2.5	50	97.5	2.5	50	97.5	2.5	50	97.5	2.5	50	97.5
1	1-3yr	0.95	1.67	2.93	0.98	1.66	9.55	0.82	1.71	2.75	0.93	1.67	2.95	0.97	1.66	9.73	0.80	1.74	2.79
2	4-6yr	0.93	1.65	2.56	0.89	1.73	2.56	0.92	1.60	2.92	0.93	2.48	2.56	0.88	1.73	2.56	0.92	1.60	2.97
3	7-10yr	0.97	1.65	2.82	0.99	1.67	2.75	0.96	1.62	2.88	0.98	1.68	2.85	0.98	1.67	2.78	0.96	1.70	2.95
4	11-18yr	0.91	1.51	2.59	1.1	1.53	5.45	0.80	1.45	2.59	0.91	1.51	2.44	1.1	1.57	5.5	0.8	1.44	2.36
5	19-29yr	0.89	1.44	2.24	0.93	1.47	2.28	0.85	1.42	2.18	0.89	1.44	2.19	0.93	1.47	2.28	0.85	1.42	2.16
6	30-50yr	0.82	1.35	2.23	0.38	1.38	2.02	0.82	1.33	5.29	0.82	1.35	2.23	0.38	1.38	2.02	0.82	1.33	5.29
7	>50	0.73	1.32	1.88	0.62	1.33	1.85	0.81	1.29	1.99	0.73	1.32	1.88	0.62	1.33	1.85	0.81	1.29	1.99

**Table 6:** Prevalence of hypothyroidism and hyperthyroidism according to age range 1-18(%)

	Overt hyperthyroidism			Subclinical hyperthyroidism			Overt hypothyroidism			Subclinical hypothyroidism		
Characteristics	Total	Men	Women	Total	Men	Women	Total	Men	Women	Total	Men	Women
Total population, categorized by age, yr.												
**All ages**	0.35	0.39	031	2.51	1.96	2.96	0.17	0.26	0.1	5.91	7.06	4.97
**1-3**	0	0	0	1.93	0.92	2.77	0.48	0.92	0	2.89	4.62	1.01
**4-6**	0.66	0.68	0.92	1.32	2.54	2.54	0.33	0	0.68	8.27	12.7	3.44
**7-10**	0.23	0.5	0	2.15	1.52	3.04	0	0	0	6.22	6.09	6.33
**11-18**	0.38	0.33	0.41	2.81	4.95	2.31	0.12	0.33	0	5.63	5.61	5.64

**Table 7:** P-values of significant statistical differences between age ranges and hormone concentration

TSH T3							T4	
Age range	4-6	7-10	1-3	4-6	7-10	11-18	1-3	4-6
11-18	-	-	≤0.001	-	-	-	-	0.007
19-29	-	0.045	≤0.001	≤0.001	≤0.001	-	-	≤0.001
30-50	-	-	≤0.001	≤0.001	≤0.001	-	-	≤0.001
>50	≤0.001	≤0.001	≤0.001	≤0.001	≤0.001	0.003	0.016	≤0.001

*Significance level is 0/05

## Discussion

 Thyroid function assessments are commonly used in the clinical chemistry. These tests are vital for disease management of the patients. TSH is an important indicator of thyroid function, and it is imperative to consider all factors affecting its determination of reference ranges. Studies report different upper and lower limits for TSH (20, 21). Because reference intervals are influenced by age, gender, race, and iodine intake, the Clinical and Laboratory Standards Institute (CLSI) and the International Federation of Clinical Chemistry (IFCC) have encouraged laboratories to set their own (8, 22) so that patients with thyroid-related diseases in different countries can receive appropriate testing and diagnosis (23, 24). We did not find any studies that offer a reference range for thyroid hormone levels in our region. As a result, our aim is to establish reference ranges for thyroid hormone concentration. In this study, for TSH, T3, and T4, the lower limits were higher, and the higher limits were lower than the reference ranges derived from the kit brochure. There is a statistical difference between hormone values after and before age 18 (*P*≤0.01). Therefore, using the same reference range for children and adults is not appropriate. Thyroid dysfunction prevalence in our study in the mentioned age population was 8.94%, and the thyroid dysfunctions in males were more prevalent than in females. 

This cross-sectional study was conducted to assess the distribution of Thyroid hormones (TSH, T3, T4) in the patients who refer to the Motahari clinic in Shiraz centre for health check-up tests in the south of Iran. In forming the reference range, age and gender are the leading factors that may influence ranges; hence our main concern in this study was whether hormone levels are related to age and sex, and it is necessary to define an age-specific range for these hormones. Many laboratories have similar reference ranges of thyroid hormones for men and women (25). Our research revealed that there were significant differences between gender only for T3 (*P*=0.03, men have a greater value of T3). So, different references should be established. We found that T4 declines with age (*P*<0.01), which agrees with other studies (26, 27). Usually, it is not necessary to establish different reference ranges of thyroid hormones based on ages in adults (28, 29), but we found that there is a statistical difference between hormone values after and before age 18 (*P*<0.001); therefore, it is not appropriate to use same reference range for children and adults. 

The prevalence of subclinical hypo and hyperthyroidism can differ based on the reference intervals of thyroid hormones. In a study in India, thyroid function abnormalities prevalence was noted in 9.18% of children and adolescents (14-19 years) (30). In our study, the total prevalence of abnormal thyroid function tests, hypo, and hyperthyroidism was 8.94%, 6.08%, and 2.86%, respectively, which is relatively similar to the study in India. Subclinical and overt hyperthyroidism prevalence in our study was 2.51% and 0.35, respectively. According to a systematic review, the subclinical and overt hyperthyroidism in all ages in our country was 1.52 and 0.69, respectively. (31). Our results indicate that hypothyroidism's prevalence was also 9.68 and 8.26 for men and women, respectively. There was male dominance in our study; however, some studies showed female dominance in hypothyroidism (32, 33). This difference might result from applying manufacturer reference range instead of laboratory range for calculating prevalence. A study in Iran also illustrates that the prevalence of thyroid disease is high in the old women population of East Azerbaijan, Iran, which desires further study in our region (34).

Small changes in reference ranges could lead to overestimation or underestimation of thyroid dysfunctions, leading to drug treatment for some patients or an increased dose for others. This simple change in reference range that has an unimagined clinical effect exemplifies the status of clear relationships at the clinical–laboratory interface. This result proposes that inter-individual variations affect the concentrations of thyroid hormones. When we set up reference ranges, age and gender may be one of these factors that should be considered.

There were several restrictions on our study. It was unfeasible to assess the alterations in thyroid function in each subject because of the essence of the study. The study included measurement values on TSH, T3, and T4, and there were no complete data on FT3 and FT4. Some data like the existence of thyroid ailment, drug history that could affect thyroid function, and a family history of thyroid dysfunctions were self-reported. 

## Conclusion

In summary, the reference range of TSH, T3, and T4 in the non-disease population was found to be more limited than that of Western countries. According to this reference range, overt and subclinical hypothyroidism prevalence was 0.17% and 5.91%, respectively. Overt and subclinical hyperthyroidism was present in 0.35% and 2.51%, respectively. 

## Ethics approval & Consent to Participate

The research was approved by the Research and Ethics Committee of the Shiraz University of Medical Sciences (Number: IR.SUMS.MED.REC.1399.509).

## Authors' contributions

MSh: Conception, design of the work, interpretation of data for the work, revising. MHI: Conception, analysis, revising. SG: Acquisition, analysis, Drafting the work. NO: Conception, design of the work, interpretation of data for the work, revising. BShY: Conception, interpretation of data for the work, revising. FA: Acquisition, analysis, Drafting the work.

## Conflict of Interest

The authors declared no conflict of interest.

## Funding

This study was funded by Shiraz University of Medical Sciences.
